# Sparse spike trains and the limitation of rate codes underlying rapid behaviours

**DOI:** 10.1098/rsbl.2023.0099

**Published:** 2023-05-10

**Authors:** Joseph M. Fabian, David C. O'Carrol, Steven D. Wiederman

**Affiliations:** ^1^ School of Biomedicine, The University of Adelaide, Adelaide, South Australia 5005, Australia; ^2^ Department of Biology, Lund University, Lund 22362, Sweden

**Keywords:** spike train, rate code, dragonfly, STMD, vision

## Abstract

Animals live in dynamic worlds where they use sensorimotor circuits to rapidly process information and drive behaviours. For example, dragonflies are aerial predators that react to movements of prey within tens of milliseconds. These pursuits are likely controlled by identified neurons in the dragonfly, which have well-characterized physiological responses to moving targets. Predominantly, neural activity in these circuits is interpreted in context of a rate code, where information is conveyed by changes in the number of spikes over a time period. However, such a description of neuronal activity is difficult to achieve in real-world, real-time scenarios. Here, we contrast a neuroscientists' *post-hoc* view of spiking activity with the information available to the animal in real-time. We describe how performance of a rate code is readily overestimated and outline a rate code's significant limitations in driving rapid behaviours.

## 1. Introduction

For sensory neural circuits, time is a valuable resource, particularly for animals that live in dynamic environments. Hunting dragonflies react to turns by prey in just 47 ms [1]. In such a short period of time, spiking neurons can only generate a small number of spikes to inform downstream control systems. Visual neurons in the motion and target-detecting pathways of flying insects have latencies in the range of 25–40 ms, usually quantified by averaging across many stimulus presentations [2–5]. However, neurons in the brain do not have the luxury of averaging technical replicates, a calculation that eliminates the sparseness and variance of spike trains. While *post-hoc* analysis of a dataset might suggest a circuit is well suited for driving rapid responses, the real-time information available to an animal could be less informative.

When a behaviour must be controlled on timescales too short for a single neuron to fire sufficient spikes, one solution is a population code that pools the outputs of multiple neurons. If these neurons have the same underlying tuning properties, the mean response of one neuron across many trials could infer an estimate of the mean population response to one individual stimulus presentation [6]. However, this assumption requires evidence that a large homogeneous cell population exists, which is not always present. For example, higher-order neurons in the dragonfly target-detecting pathway appear to be heterogeneous in terms of receptive field location, direction tuning and spiking statistics [7–11]. In such a system, pooling across a population of neurons may occur, but many repetitions from a single neuron will produce a false estimate of real-time population activity. This averaging calculation causes overestimation of rate code performance.

In this paper, we use recordings from the dragonfly visual system to evaluate the limitations of implementing an effective rate code in the target detection pathway. We highlight differences between the view of an experimenter and an organism, noting that this conflation has caused researchers to overestimate the performance of neuronal circuits.

## 2. Material and methods

We recorded intracellularly from the centrifugal small target motion detector (CSTMD1) neuron [8] in 24 immobilized wild-caught dragonflies (*Hemicordulia tau*) while presenting small moving dark targets (2° × 2°) on a 165 Hz monitor. Test targets appeared within the receptive field and drifted at 80° s^−1^. CSTMD1 responses are facilitated by targets that move on long continuous trajectories, so we also included a ‘primed' condition for comparison. In primed trials, the test target trajectory was preceded by a 500 ms target trajectory terminating at our test target location, either 150 or 300 ms prior to our test target appearance. These stimuli were chosen because they stimulate STMD neurons optimally [12] allowing us to view spiking data from a best-case-scenario condition. Data were sampled at 5000 Hz and analysed offline in MATLAB.

Examples of quantifying neural activity were generated from CSTMD1's responses to 41 repetitions of an identical drifting target in one dragonfly (only 20 repetitions displayed in raster plots for clarity). Peristimulus time histograms (PSTHs) were produced by binning spikes into 20 ms bins. To define CSTMD1's response latency to the presentation of a stimulus, we adapted a noise-based, thresholding technique by Warzecha & Egelhaaf [2]. This approach is sensitive to the distribution of spontaneous spike rates, so we pooled data from 1008 recordings of 1-s long of CSTMD1's spontaneous activity (across 12 dragonflies). We determined a spike rate threshold equal to two standard deviations above this mean spontaneous activity. Latency was defined as the first time mean activity surpassed this threshold (1 ms bins, 432 target presentations in the same 12 dragonflies). We also generated cumulative spike count plots, spike rate plots and spike burst plots from the same set of trials, by determining the proportion of trials which produced a given output at each point in time.

We described theffi distribution of spontaneous spiking intervals from 2500 spikes from CSTMD1 in one dragonfly. To investigate temporal patterns in spontaneous activity we recorded 19 635 sponaneously fired spikes across 24 dragonflies. We determined the correlation of intervals between each spike and the second through 10th subsequent spikes.

## 3. Results

### (a) Quantifying neural responses to stimuli

**Figure 1.**
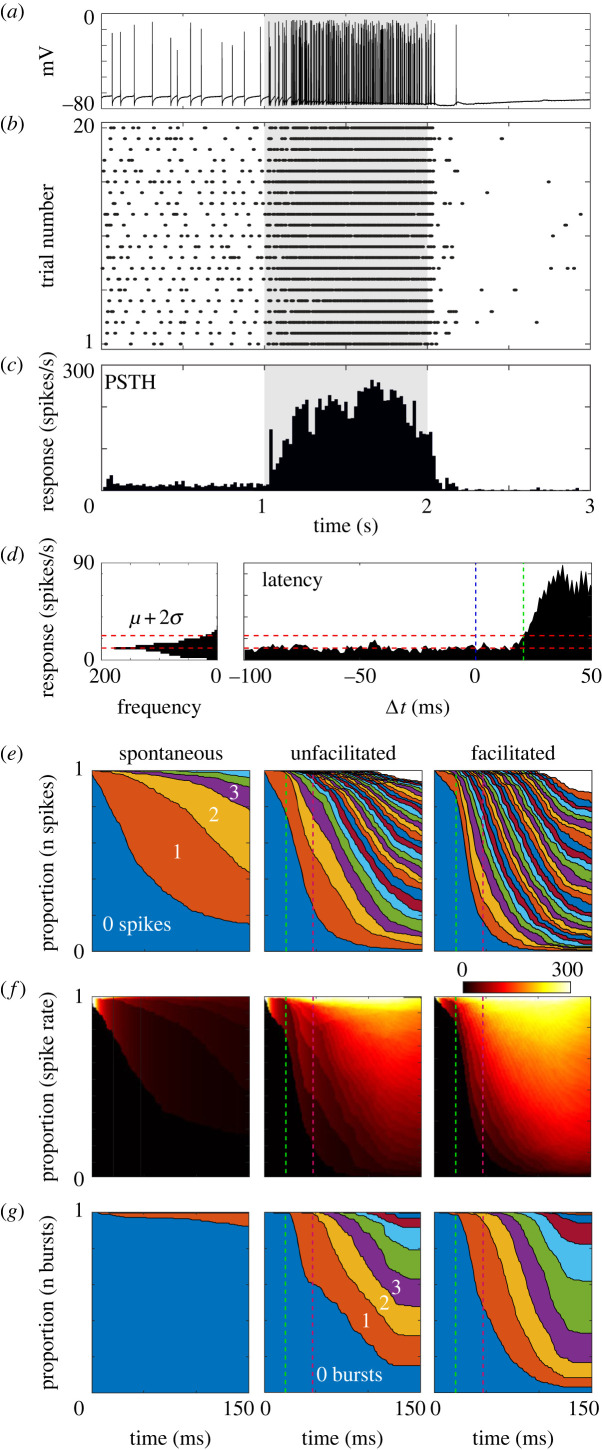
Quantifying neuronal responses in visual neurons. (*a*) An example intracellular recording from CSTMD1 during the presentation of a moving target (grey-shaded region indicates peristimulus duration). (*b*) Spike raster displaying 20 presentations of an identical stimulus from the same neuron. (*c*) Peristimulus time histogram (PSTH, 20 ms bins) computed from the spiking data in (*b*). (*d*) Calculating the response latency with the approach of [2] (432 trials from 12 dragonflies). (*e*) The same data as (*d*), displaying the proportion of trials with a given cumulative spike count as a function of time, in the absense (left) and presence of either an unfacilitated (middle) or facilitated (right) moving target stimulus. Green and magenta dashed lines indicate the 21 ms latency from (*d*), and the 47 ms behavioural delay from [1]. (*f*) The equivelant spike rate from (*d*). (*g*) Displaying the proportion of trials with a given cumulative burst count as a function of time.

We recorded intracellular activity from CSTMD1 while presenting a series of moving targets. Following presentation of a target, spiking activity increases (figure 1*a*). If we repeat this stimulus in the same neuron, the pattern of spikes is similar but not identical (figure 1*b*). Individual variations in spiking activity between trials are often removed by pooling responses across technical replicates. The mean response of a neuron is commonly displayed by a PSTH, where spikes from all trials are binned according to their time (20 ms bins), and the spike count in each bin is converted to instantaneous spike rates (figure 1*c*).

When a dragonfly pursues prey, the temporal latency between stimulus presentation and neural response is critical. For neurons that lack spontaneous activity, this can be defined as the arrival time of the first spike. In neurons that fire spontaneously, the latency becomes a statistical problem, as to when responses differ significantly from the pre-stimulus condition, e.g. by surpassing a threshold based on a spike distribution of spontaneous activity (dashed red lines, figure 1*d*, *N* = 12 dragonflies). The first time the mean response to moving targets surpasses this threshold can be defined as the latency (dashed green line).

These laboratory measures exist *post-hoc*, but they do not exist in a biological pursuit. These analytical approaches may be valuable for our understanding of neuronal circuits, however they address a fundamentally different problem to how a neuronal circuit interprets real spike trains. To demonstrate this issue, we used the same dataset in figure 1*d* and determined the cumulative number of spikes at each time point in each trial (figure 1*e*). In the absence of a stimulus (i.e. spontaneous activity), spikes slowly accumulate over time. When a target is presented (i.e. unfacilitated), spikes accumulate much faster, and when that target is preceded by a continuous target trajectory [3,12,13] facilitation further accelerates responses. From these data we calculated an increase in spike rate after 21 ms. However, at this point in time 76% of trials contained no new spikes, and only 1.4% of trials had two or more spikes, the minimum requirement to define a stimulus-specific change in rate (green dashed line). We can also calculate the distribution of observed spike rates from the same data (figure 1*f*). Spike rates remain very low and heavily quantized for the majority of trials until well beyond the reported neural latency of 21 ms. CSTMD1 fires spikes in a series of bursts rather than regular single spikes [11]. Plotting the timing of spike bursts from the same data highlights that a burst code removes most spontaneous activity (figure 1*g*). However by the fundamental nature of bursts, they are significantly more sparse than individual spikes. In most cases, 21 ms following the presentation of a target, CSTMD1 can contribute little to nothing to real-time control of behaviour.

During pursuits, dragonflies respond on average to prey movements in just 47 ms [1] (magenta dashed line). Even if we assume that all processing stages following STMD neurons are instantaneous, 47 ms following stimulus onset only 53% of trials contain two or more spikes. The event of two spikes in a 47 ms period also occurred in 5% of spontaneous trials. If two spikes are insufficient to drive behaviour, then most trials will result in false negatives (i.e. missing a target's presence). Alternatively, if two spikes are sufficient, a false-positive signal from spontaneous activity will occur every 940 ms of a dragonfly's life (on average). Either case produces an undesirable outcome.

### (b) The problem of spontaneous activity

**Figure 2.**
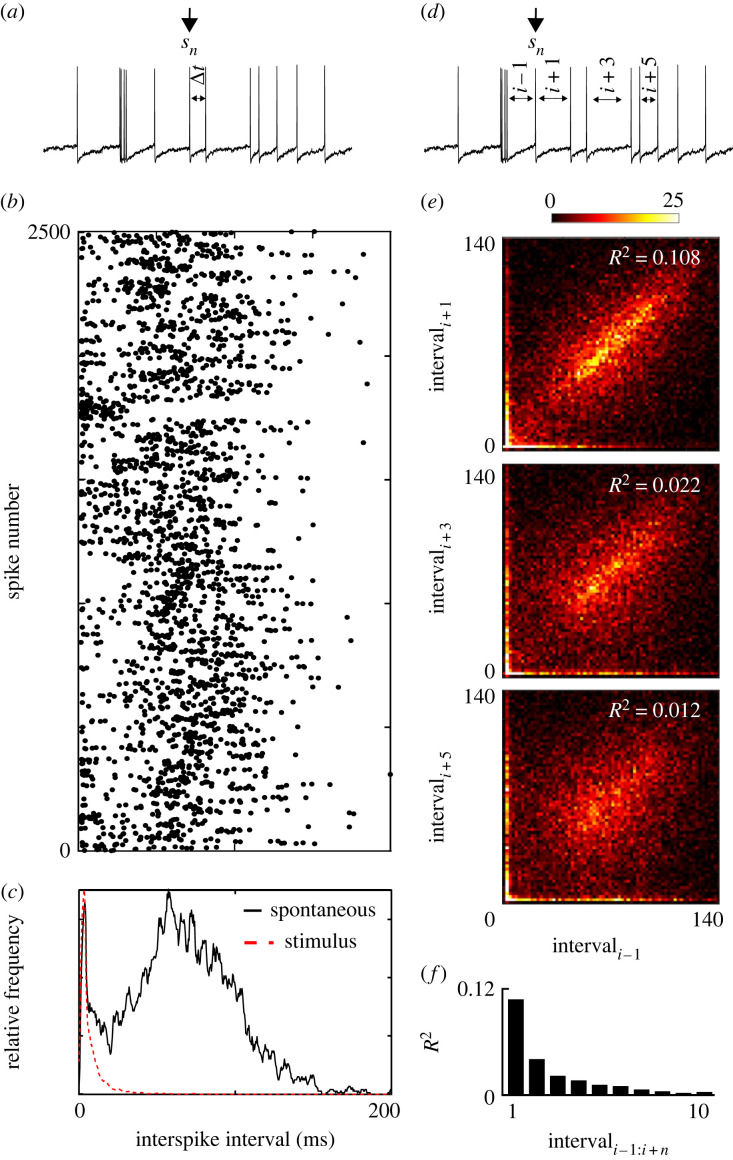
Spontaneous activity and the lack of regularity in spike timing. (*a*) A raw train of CSTMD1's spontaneous spiking activity. We compute the time interval between each spike. (*b*) The distribution of spike intervals from 2500 spontaneous spikes in a single CSTMD1 recording. (*c*) A histogram of the spontaneous interspike intervals shown in (*b*) (black), compared to stimulus-generated interspike intervals (dashed red). (*d*) The approach for correlating spontaneous spike intervals. (*e*) The previous interval (*i*:*i*−1) plotted against the next interval (*i*:*i* + 1), the third next interval, and the fifth next interval for 19 635 spontaneously generated spikes across 24 dragonflies. (*f*) Spike intervals are poorly correlated and this correlation rapidly drops further along the spike train.

Many neurons, including CSTMD1, fire action potentials at low rates in the absence of a stimulus [8]. To be detectable, a stimulus must drive responses significantly higher than this background spontaneous activity (as described in figure 1). Research often reports mean spontaneous spike rates over large windows of time and compare this with the mean responses to a stimulus [3,12]. However, neurons do not output mean spike rates, rather they output spikes which stochastically generate the release of neurotransmitter for downstream processing. Since spontaneous and stimulus driven spikes are identical at behaviourally relevant time periods, they can only be distinguished by their temporal patterns. We recorded prolonged periods of spontaneous activity in an individual CSTMD1 (figure 2*a*) and calculate spike intervals to evaluate the temporal statistics. We plot the distribution of interspike intervals in spontaneous activity, and compare to intervals observed during responses to stimuli (figure 2*b*). We see that CSTMD1's spontaneous activity contains a broad distribution of spike intervals (figure 2*c*), including a significant peak at intervals shorter than 5 ms. To determine how rapidly spike intervals in spontaneous activity change, for 19 635 spontaneous spikes across 24 dragonflies, we plot the interval for the previous spike (*i* − 1) against the interval for the next spike (*i* + 1), the third next spike, and the fifth next spike (figure 2*d,e*). Intervals separating immediately neighbouring spikes are only weakly correlated (*R*^2^ = 0.12), and this correlation quickly drops for successive spikes in a train (figure 2*f*). This data show that spontaneous activity is very broad, contains many short spike intervals, and is weakly correlated in time. Therefore over short behaviourally relevant timescales a spontaneous rate is not readily defined, and distinguishing the differences between spontaneous and stimulus generated activity is challenging.

## 3. Discussion

Our understanding of brain function relies on an accurate measure and interpretation of neural activity. The sparse spike trains that sensory neurons generate can be quantified and interpreted in varying frameworks, including rate or temporal codes. However, decoding spike trains into a representative signal is not trivial, inducing significant temporal processing delays, particularly troublesome in the context of sensorimotor latencies. We reiterate findings from previous work which demonstrate that technical and biological replication of data eliminates key issues facing neural circuits, causing overestimation of performance [6,14–16]. Spike rates in individual trials may share little resemblance with the mean PSTH, to the point where simply distinguishing responses from spontaneous activity becomes difficult on the timescales dragonfly behaviours are controlled. The data we have presented here represents a best case scenario, because we compare responses to an optimal stimulus to no stimulus at all. If the role of STMDs was more nuanced than simply reporting the presence of a stimulus, but its velocity, position or direction, the described challenges would be magnified.

Here we have demonstrated that the ultimate limitating factor for target detection is the lack of spikes on short timescales. Even in the best case scenario with optimal, fully facilitated stimuli, by 47 ms more than 50% of trials contained three or fewer spikes. It is possible that in natural conditions these neurons fire at higher rates, but the responses described here remain stronger than most visual neurons studied. The responses of visual neurons in insects are almost universily defined by their spike rates, but this sparseness renders a rate code of little use on short timescales. Reporting spike rate is popular because it is both simple and versatile, but by its own definition it ignores potentially critical temporal information in spike trains. Since multiple spikes are required to define a rate, and spikes can be separated by tens or even hundreds of milliseconds, rate codes are inherently sluggish [14]. When considering sensorimotor control of rapid behaviors, this is particularly troublesome.

Given the limitations of rate coding, how are rapid behaviours controlled from small populations of neurons? We know that whatever solutions dragonflies use, it is remarkably effective. Spike timing or bursting codes can permit faster reaction times than rate codes, because the relevant signal is contained in fewer spikes. In systems that require rapid response readouts, the timing of the first spike might be the information source [15,16]. However due to the relatively high spontaneous spike rates in the STMD system, the first spike following stimulus onset is spontaneously generated in approximately 68% trials, so such a system would not work well. Under the right conditions small numbers of spikes in individual neurons can still encode useful information about the outside world [6,17]. Every spike or absence of spike in a spike train alters the distribution of possible external stimuli. However for a neuron that fires more than 13 spikes per second spontaneously, an individual spike will have a very small impact on this distribution. In the presence of this spontaneous activity, multiple spikes need to occur within a short time period in order to signal a high likelihood of a targets presence. Recent work found that when a dragonfly is presented with a salient stimulus, CSTMD1 fires spikes in a series of bursts [11]. Bursting presents significant signal-to-noise ratio benefits [18], especially in CSTMD1 where bursts are extremely rare in spontaneous activity. However because the primary temporal limitation is the overall lack of spikes on short timescales, all coding strategies which rely on individual neuron activity will tend to perform poorly.

Given that individual neurons fire so few spikes, the most reasonable solution to encode information is through a population code which pools activity from the heterogeneous STMD population. Only 3 large-field STMD neurons have been characterized to date (CSTMD1, CSTMD2 and BSTMD1), but there are many other uncharacterized STMD neurons in the dragonfly lobula which could form part of a population code. Heterogenous populations might actually be advantageous for a population code, because heterogeneosity reduces correlation across a population which improves information content [19]. The study of STMD neurons is heavily skewed by the fact that only a small number of cells have large axons which facilitate electrophysiological recording. Until we are able to build a more comprehensive database of the STMD population, it is difficult to propose a realistic target detecting model.

The issues highlighted here are not alleviated downstream from STMDs, in fact they are greatly magnified. Target selective descending neurons (TSDNs), exhibit similar tuning but fire at far lower rates than their upstream STMD counterparts [4,20,21]. According to recent work, when presented targets moving on 100 ms trajectories, 43 of 44 TSDNs studied fired less than one spike per trajectory [4]. There are only eight of these neurons on each side of the brain, and each neuron has different direction tuning and receptive field. Pooling across a population is impossible because only one of each cell type exists, and pooling across time to sample sufficient spikes would require several seconds of strong stimulation. Even if the activity of all eight cells were pooled together, 47 ms of target motion would contain approximately 0.09 spikes on average. On the timescales that dragonfly behaviours take place, the dragonfly target detecting circuits do not fire enough spikes. To understand the control of rapid and accurate behaviours, future work must study spike timing in detail, and focus on distinguishing practical measures of neural activity from realistic neural codes and control systems.

## Data Availability

The data are provided in the electronic supplementary material [22].

## References

[1] Mischiati M, Lin HT, Herold P, Imler E, Olberg R, Leonardo A. 2015 Internal models direct dragonfly interception steering. Nature **517**, 333-338. (10.1038/nature14045) 25487153

[2] Warzecha AK, Egelhaaf M. 2000 Response latency of a motion-sensitive neuron in the fly visual system: dependence on stimulus parameters and physiological conditions. Vis. Res. **40**, 2973-2983. (10.1016/S0042-6989(00)00147-4) 11000395

[3] Nordström K, Douglas M, Bolzon DM, O'Carroll DC. 2011 Spatial facilitation by a high-performance dragonfly target-detecting neuron. Biol. Lett. **7**, 588-592. (10.1098/rsbl.2010.1152) 21270026 PMC3130215

[4] Gonzalez-Bellido PT, Peng H, Yang J, Georgopoulos AP, Olberg RM. 2013 Eight pairs of descending visual neurons in the dragonfly give wing motor centers accurate population vector of prey direction. Proc. Natl Acad. Sci. USA **110**, 696-701. (10.1073/pnas.1210489109) 23213224 PMC3545807

[5] Supple JA et al. 2020 Binocular encoding in the damselfly pre-motor target tracking system. Curr. Biol. **30**, 645-656. (10.1016/j.cub.2019.12.031) 31956029

[6] Rieke F, Warland D, De Ruyter van Steveninck R, and Bialek W. 1997 Spikes: exploring the neural code. Cambridge, MA: MIT press.

[7] O'Carroll DC. 1993 Feature detecting neurons in dragonflies. Nature **362**, 541-543. (10.1038/362541a0)

[8] Geurten BRH, Nordström K, Sprayberry JDH, Bolzon DM, O'Carroll DC. 2007 Neural mechanisms underlying target detection in a dragonfly centrifugal neuron. J. Exp. Biol. **210**, 3277-3284. (10.1242/jeb.008425) 17766305

[9] Dunbier JR, Wiederman SD, Shoemaker PA, O'Carroll DC. 2012 Facilitation of dragonfly target-detecting neurons by slow moving features on continuous paths. Front. Neural Circuits. **6**, 79. (10.3389/fncir.2012.00079) 23112764 PMC3483020

[10] Evans BJE, Fabian JM, O'Carroll DC, Wiederman SD. 2022 Dragonfly neurons selectively attend to targets within naturalistic scenes. Front. Cell. Neurosci. **16**, 857071. (10.3389/fncel.2022.857071) 35450210 PMC9017788

[11] Fabian JM, Wiederman SD. 2021 Spike bursting in a dragonfly target-detecting neuron. Sci. Rep. **11**, 1-6. (10.1038/s41598-020-79139-8) 33597665 PMC7889644

[12] Wiederman SD, Fabian JM, Dunbier JR, O'Carroll DC. 2017 A predictive focus of gain modulation encodes target trajectories in insect vision. Elife **6**, e26478. (10.7554/eLife.26478) 28738970 PMC5526664

[13] Fabian JM, Dunbier JR, O'Carroll DC, Wiederman SD. 2019 Properties of predictive gain modulation in a dragonfly visual neuron. J. Exp. Biol. **222**, jeb207316. (10.1242/jeb.207316) 31395677

[14] Brette R. 2015 Philosophy of the spike: rate-based vs. spike-based theories of the brain. Front. Syst. Neurosci. **9**, 151. (10.3389/fnsys.2015.00151) 26617496 PMC4639701

[15] VanRullen R, Guyonneau R, Thorpe SJ. 2005 Spike times make sense. Trends Neurosci. **28**, 1-4. (10.1016/j.tins.2004.10.010) 15626490

[16] Shamir M. 2014 Emerging principles of population coding: in search for the neural code. Curr. Opin Neurobiol. **25**, 140-148. (10.1016/j.conb.2014.01.002) 24487341

[17] Fairhall AL, Lewen G, Bialek W, van Stevenick R. 2001 Multiple timescales of adaptation in a neural code. Nature **412**, 787-792. (10.1038/35090500) 11518957

[18] Krahe R, Gabbiani F. 2004 Burst firing in sensory systems. Nat. Rev. Neurosci. **5**, 13-23. (10.1038/nrn1296) 14661065

[19] Chelaru MI, Dragoi V. 2008 Efficient coding in heterogeneous neuronal populations. Proc. Natl Acad. Sci. USA **105**, 16 344-16 349. (10.1073/pnas.0807744105) PMC2571028 18854413

[20] Olberg RM. 1986 Identified target-selective visual interneurons descending from the dragonfly brain. J. Comp. Physiol. A **159**, 827-840. (10.1007/BF00603736)

[21] Frye M, Olberg R. 1995 Visual receptive field properties of feature detecting neurons in the dragonfly. J. Comp. Physiol. A **177**, 569-576. (10.1007/BF00207186)

[22] Fabian JM, O'Carroll DC, Wiederman SD. 2023 Sparse spike trains and the limitation of rate codes underlying rapid behaviours. Figshare. (10.6084/m9.figshare.c.6620278) PMC10170213 37161293

